# Similar Comparative Low and High Doses of Deltamethrin and Acetamiprid Differently Impair the Retrieval of the Proboscis Extension Reflex in the Forager Honey Bee (*Apis mellifera*)

**DOI:** 10.3390/insects6040805

**Published:** 2015-09-28

**Authors:** Steeve H. Thany, Céline M. Bourdin, Jérôme Graton, Adèle D. Laurent, Monique Mathé-Allainmat, Jacques Lebreton, Jean-Yves Le Questel

**Affiliations:** 1 LBLGC UPRES EA 1207, Collégium Sciences et Techniques, Université d’Orléans, 2 rue de Chartres, BP 6759, Orléans 45067, France; E-Mail: celine.bourdin@univ-angers.fr (C.M.B.); 2 RCIM UPRES EA 2647, USC INRA 1330, Université d’Angers, 2 Bd. Lavoisier, Angers 49045, France; 3 CEISAM UMR 6230, UFR des Sciences et des Techniques, Université de Nantes, 2 rue de la Houssinière, BP 92208, Nantes F-44000, France; E-Mails: jerome.graton@univ-nantes.fr (J.G.); adele.laurent@univ-nantes.fr (A.D.L.); monique.mathe@univ-nantes.fr (M.M.-A.); jacques.lebreton@univ-nantes.fr (J.L.); jean-yves.le-questel@univ-nantes.fr (J.-Y.L.Q.)

**Keywords:** Neonicotinoid, pyrethroid, pesticides, acetamiprid, deltamethrin, honey bee, insect

## Abstract

In the present study, the effects of low (10 ng/bee) and high (100 ng/bee) doses of acetamiprid and deltamethrin insecticides on multi-trial learning and retrieval were evaluated in the honey bee *Apis mellifera*. After oral application, acetamiprid and deltamethrin at the concentrations used were not able to impair learning sessions. When the retention tests were performed 1 h, 6 h, and 24 h after learning, we found a significant difference between bees after learning sessions when drugs were applied 24 h before learning. Deltamethrin-treated bees were found to be more sensitive at 10 ng/bee and 100 ng/bee doses compared to acetamiprid-treated bees, only with amounts of 100 ng/bee and at 6 h and 24 h delays. When insecticides were applied during learning sessions, none of the tested insecticides was able to impair learning performance at 10 ng/bee or 100 ng/bee but retention performance was altered 24 h after learning sessions. Acetamiprid was the only one to impair retrieval at 10 ng/bee, whereas at 100 ng/bee an impairment of retrieval was found with both insecticides. The present results therefore suggest that acetamiprid and deltamethrin are able to impair retrieval performance in the honey bee *Apis mellifera*.

## 1. Introduction

The beekeeping industry represents several billion dollars in the world due to the importance of bees in agricultural crop pollination, natural ecosystems, and plant biodiversity [1]. In this context, there is great concern about the decline of the honey bee *Apis mellifera* in several parts of the world. The collapse of honey bee colonies (CCD: colony collapse disorder) in developed countries (Europe and America) has focused research on two main fronts—biological factors like viruses or parasites [2], and pesticide contamination [3,4]—because these countries have a long history of using pesticides in agriculture. Among the pesticides, insecticides such as pyrethroids and the newly developed neonicotinoids have been targeted as the main ones involved in CDD [5]. For example, it was demonstrated that honey bees exposed to nonlethal doses of neonicotinoids could result in homing failure [6]. In general, it was suggested that low levels of pesticides may acts as stressors and/or may result in an impairment of learning and memory processes. Thus, in addition to being a system model for studying olfactory learning and memory formation [7,8,9,10], bees remained a model for understanding the toxicological effects and mode of action of pesticides [11,12,13,14,15].

In forager bees, a learning process associates floral parameters such as odor and color of flowers with food reward. Thus, the conditioning of proboscis extension reflex (PER) has given fundamental results leading us to study the effect of pesticides on learning and memory in honey bees. Imidacloprid (IMI), the leading compound of neonicotinoid insecticides, was used to demonstrate the ability of neonicotinoids to decrease bee performance and learning ability [12,16]. The results from IMI were confirmed by testing other pesticides. Two compounds in particular, deltamethrin (DEL) and acetamiprid (ACT), were used to test the effects of pyrethroids and neonicotinoids on learning and memory. Previous studies demonstrated that DEL at 500 µg.kg^−1^ induces a decrease in both the foraging activity on the food source and activity at the hive entrance [16]. Interestingly, DEL did not induce an impairment on learning performances of restrained individual bees [16]. Using ACT against emergent bees, it was demonstrated that following oral application, its only significant effect was an increase in responsiveness to water at 0.1 µg. Indeed, it was shown that ACT given orally induced an increase in water responsiveness at 1 h and 3 h and had no effect on learning sessions (five conditioning trials), despite the fact that all bees gained 60% in conditioning performance [17]. These results seemed to demonstrate no specific effect of either DEL or ACT on learning and memory performance in the honey bee *Apis mellifera*.

In the present study, the toxicological effects of the neonicotinoid insecticide ACT and the pyrethroid DEL on learning and memory processes are compared using the same concentrations and conditions. The concentrations used were tested according to previously published studies [12,14,17]. The results are interpreted in relation to previous data and experimental conditions.

## 2. Materials and Methods

### 2.1. Animals

Honey bees (*Apis mellifera*) were caught in flowers, close to the hive, in the University of Orléans garden. At the laboratory, they were anaesthetized by freezing and individually fixed in small plastic tubes with a drop of wax-rosin mixture on the dorsal part of the thorax and the head. The antennae and mouth parts were free to move. After being fed with 40% sucrose, honey bees underwent a 3 h food deprivation period before the experiment started. For each bee, the experiment lasted for two days. Animals were restrained throughout the experiment and fed to satiation with the same sucrose solutions (constant volume) at the end of the first day to survive until the second day. During the experiment we use only summer honey bees.

### 2.2. Exposure Protocols

**Figure 1:**
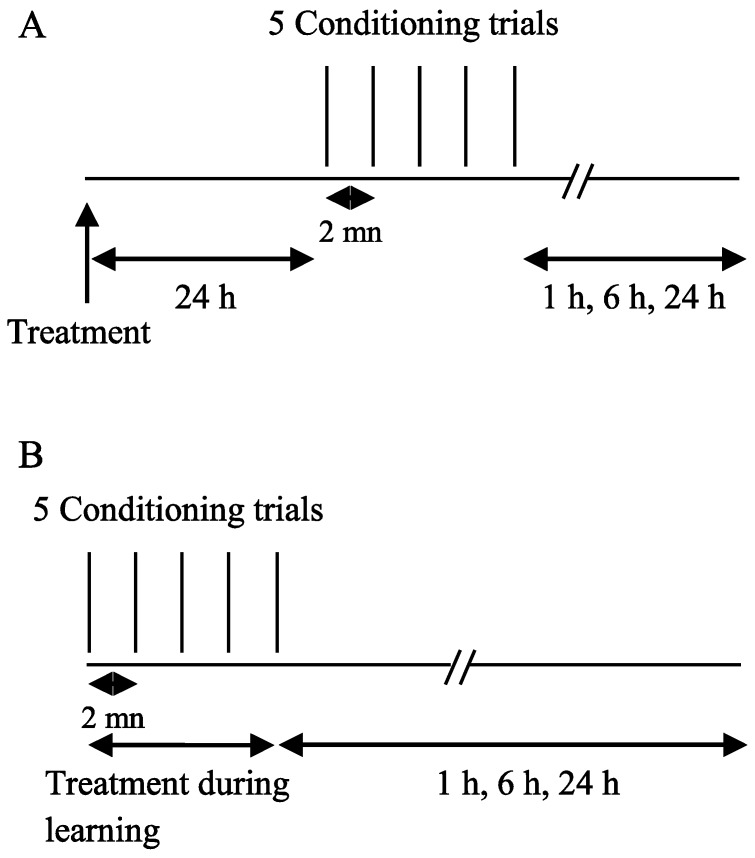
Behavioral paradigm used to evaluate the effects of ACT and DEL on learning and retrieval in the forager bees. (**A**) Drugs were orally applied 24 h before learning sessions; (**B**) bees were fed with drugs during learning sessions and then the retrieval was evaluated at 1 h, 6 h, and 24 h after learning sessions.

**Figure 2:**
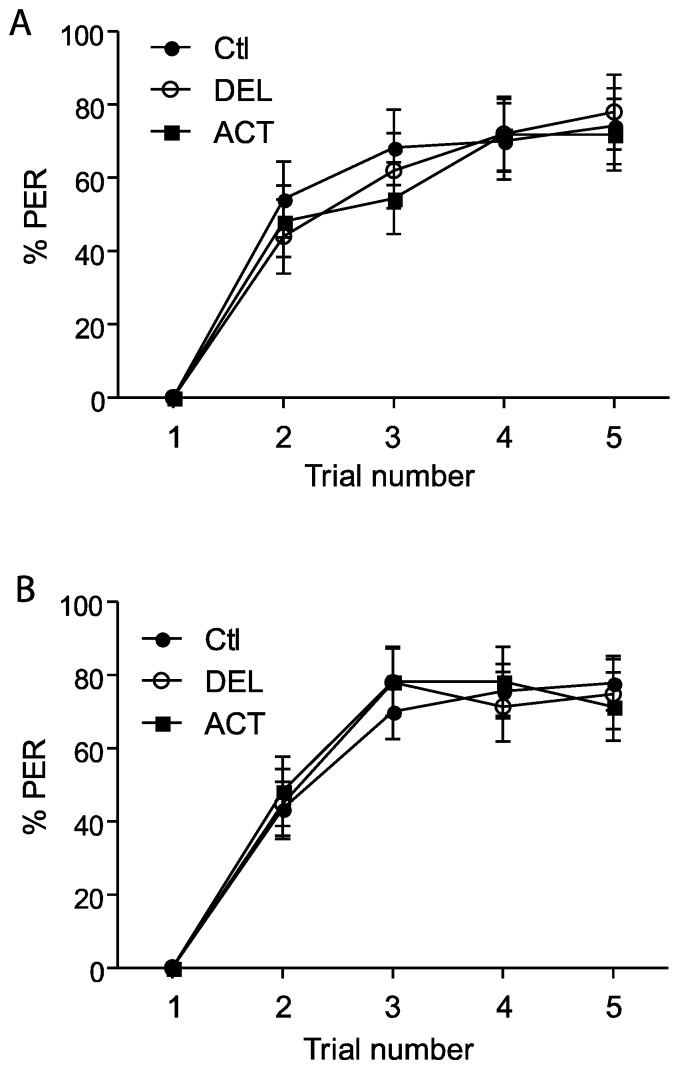
PER responsiveness of honey bees when drugs were applied 24 h before learning sessions. (**A**,**B**) bees treated with 10 ng or 100 ng of ACT or DEL, respectively. The learning performance is expressed as the percentage of PER to antennal stimulation with 40% sucrose solution. Each group represents n = 90 bees. Bars represent standard errors. Acetamiprid (ACT), deltamethrin (DEL), and control groups (Ctl).

Two oral exposure protocols were used (Figure 1): (1) forager bees were fed with ACT, DEL, or sucrose solution 24 h before learning sessions; the retention sessions were tested at 1 h, 6 h, and 24 h after learning (Figure 1A); (2) forager bees were fed with drugs or sucrose during the five learning trials and then the retrieval was estimated 1 h, 6 h, and 24 h after learning trials (Figure 1B).

### 2.3. Drugs

DEL and ACT were purchased from SIGMA (Saint Quentin Fallavier, France). They were used at doses of 10 ng/bee and 100 ng/bee but, contrary to previous studies, they were first dissolved in DMSO to achieve a final concentration of 0.1% and afterwards in 40% sucrose solution [17]. These were applied orally using a syringe.

### 2.4. Behavioral Experiments

The olfactory conditioning of the proboscis extension response (PER) in the honey bee consists of temporal pairing of an odor (lavender oil, conditioned stimulus, CS) with sugar stimulation of the antennae and proboscis (unconditioned stimulus, US), which induces the PER (unconditioned response, UR). After training, the CS presentations alone are able to elicit the PER (conditioned response, CR). The lavender oil was diffused from a piece of filter paper placed in the syringe and renewed daily.

The olfactory stimulation (or CS) was directed toward the antennae for 6 s and diffused through an air puff by means of a 5-mL syringe. Three seconds after odor onset, both antennae were touched for 6 s with a drop of sugar water (sucrose solution, 40%; US). Hence, there was an overlap of 3 s between the CS and the US. The sucrose stimulation elicited the PER. The forager honey bees were allowed to drink the sucrose solution for 3 s. Five CS/US pairing trials were applied following this procedure with an inter-trial interval of 2 min. The retrieval tests consisted of presentations of the CS alone. The rate of bees showing the conditioned response was used as a measure for the successful association between CS and US. For each bee retrieval was tested 1 h, 6 h, and 24 h after the last learning trial [17,18].

The retrieval level in each group was evaluated as the CR rate (see results for details). At the end of each experiment, the UR to antennal sugar stimulation was tested in those honey bees that did not respond to the odor presentation during the retention tests. This was done to be sure that the treatments did not impair the motor component of the proboscis extension or the sucrose perception. Only bees which showed UR were included in the study.

### 2.5. Statistical Analyses

In all our experiments, the graphs and histograms show the PER rate, which is the proportion of honey bees displaying a PER to the odor during a retrieval test or a learning trial. The sample size for each group was 90 bees. The results were analyzed using R 2.0 (R Development Core Team, University of California, Los Angeles, CA. USA, 2004). All tests were two-tailed and the significance level was set to 0.050. As we have binary data, Fisher’s exact tests were computed to compare the PER rate between the different groups.

## 3. Results

### 3.1. Effect of ACT and DEL on Acquisition

**Figure 3:**
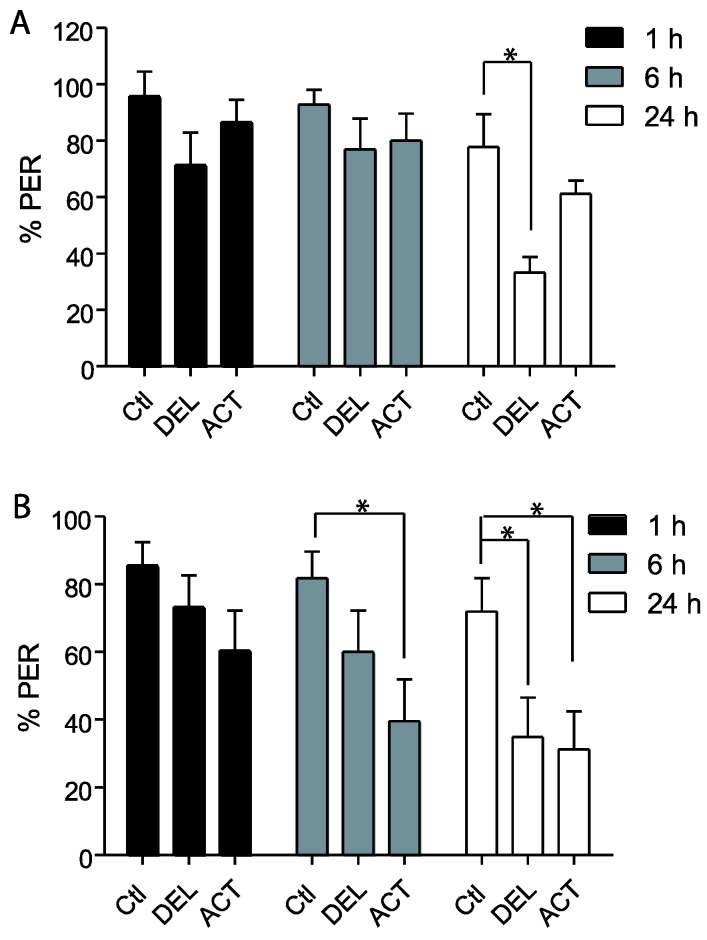
Retrieval responsiveness of honey bees when insecticides are applied 24 h before learning sessions. (**A**,**B**) bees treated with 10 ng/bee or 100 ng/bee of ACT or DEL, respectively. Data are expressed as the percentage of PER performance to antennal stimulation with 40% sucrose. Significant results are indicated by * *p* < 0.05. Each group represents n = 90 bees. Bars represent standard errors. Acetamiprid (ACT), deltamethrin (DEL), and control groups (Ctl).

In the first experiments, drugs were applied 24 h before the conditioning of the PER and the retrieval was tested at 1 h, 6 h, and 24 h after conditioning (See Figure 1A). After oral intoxication, ACT and DEL did not alter the learning sessions (Figure 2). Similar results were found at both 10 ng (Figure 1A) and 100 ng (Figure 1B) concentrations. These trends indicate that ACT and DEL have no effect on learning performance. No statistical significant differences were found between control and tested forager bees (*p* > 0.005). At the low and high doses tested, both ACT and DEL appear therefore not to be able to disturb learning performance. Interestingly, looking at retrieval performance, it appeared that at 10 ng/bee, DEL disturbed retrieval of PER at 24 h after conditioning. Significantly different results were found between DEL-treated bees (*p* = 0.0072, n = 90, Figure 3A) and control groups. Similar results were also demonstrated at 100 ng/bee with DEL at 24 h (*p* = 0.0087, n = 90, Figure 3B) and ACT at both 6 h (0.024, n = 90, Figure 3B) and 24 h (*p* = 0.030, n = 90, Figure 3B) after learning sessions. These results suggest that at a lower dose, DEL is more toxic than ACT on retrieval performance despite the fact that we found a decrease in PER responses for ACT compared to control groups, at 24 h (no statistical difference, see Figure 3A).

### 3.2. Effect of ACT and DEL Applied during Learning Trials of the PER

**Figure 4:**
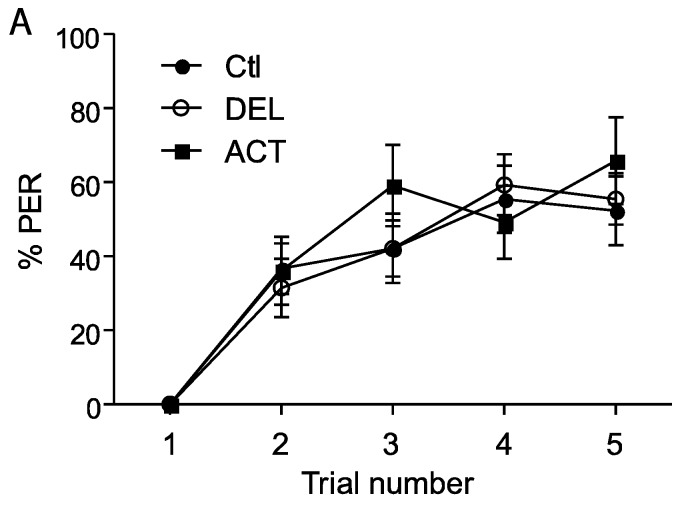

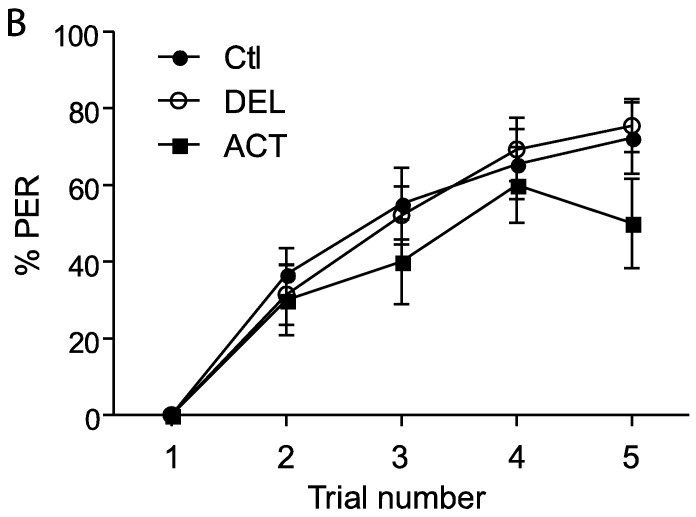
PER responsiveness of honey bee when drugs were applied during learning sessions. (**A**,**B**) bees treated with 10 ng/bee or 100 ng/bee of ACT or DEL, respectively. The learning performance is expressed as the percentage of PER to antennal stimulation with 40% sucrose solution. Each group represents n = 90 bees. Bars represent standard errors. Acetamiprid (ACT), deltamethrin (DEL), and control group (Ctl).

**Figure 5:**
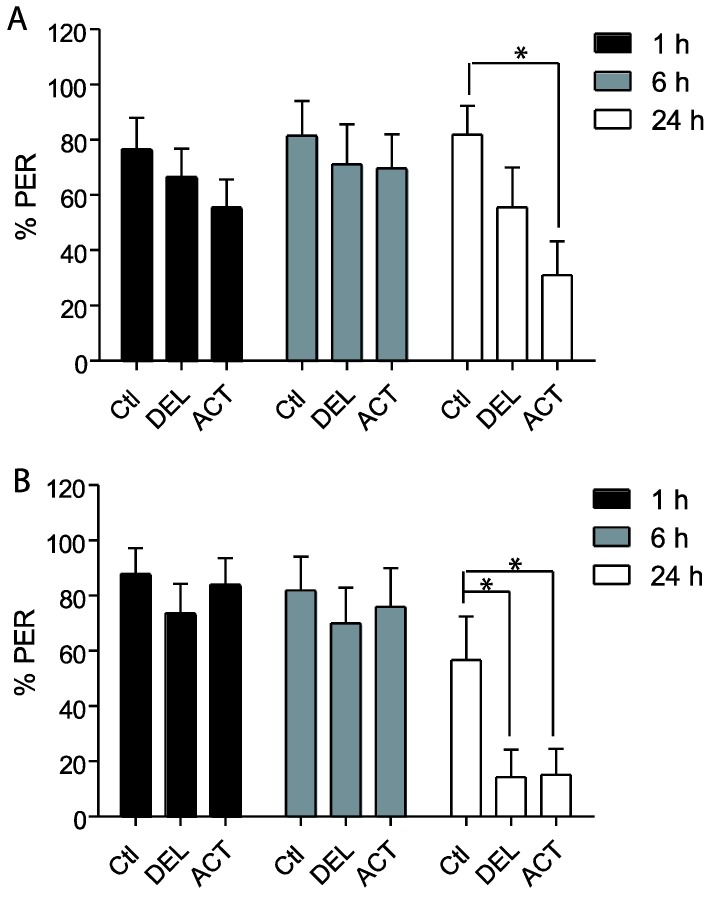
Retrieval responsiveness of honey bees when drugs are applied during learning sessions. (**A**,**B**) bees treated with 10 ng/bee or 100 ng/bee of ACT or DEL, respectively. Data are expressed as the percentage of PER performance to antennal stimulation with 40% sucrose. Significant results are indicated by * *p* < 0.05. Each group represents n = 90 bees. Bars represent standard errors. Acetamiprid (ACT), deltamethrin (DEL), and control groups (Ctl).

The retrieval test performed 24 h after the learning sessions showed a significant decrease of PER performance after oral application of ACT and DEL. Consequently, bees were fed with ACT and DEL during learning sessions and retrieval were again evaluated at 1 h, 6 h, and 24 h after learning (see Figure 1A). In these conditions, no significant effect on learning session was found for both compounds (Figure 4A,B). As previously demonstrated, ACT and DEL are not able to impair learning performance but they were able to impair retrieval at 24 h after learning (Figure 5). At 10 ng/bee, a significant decrease in performance was found with ACT (*p* = 0.009, n = 90, Figure 5A). The retrieval test performed at 100 ng/bee demonstrated a strong effect on both ACT- (*p* = 0.028, n = 90, Figure 5B) and DEL-treated bees (*p* = 0.023, n = 90, Figure 5B). These results were consistent with a strong effect of ACT and DEL at higher doses.

## 4. Discussion

This study aims to compare, at the same concentrations and conditions, the effects of two pesticides, ACT and DEL. When they were applied 24 h before or during learning, we found that they did not affect learning sessions. These data are in accordance with previous studies demonstrating that DEL did not affect learning performance in the worker honeybee [12]. Similar results were found with ACT, indicating no strong effect on learning performance at high and low doses. When drugs were applied 24 h before learning sessions, ACT appeared more effective than DEL at 24 h after learning sessions (48 h after drug application), suggesting that the apparent lack of effect on learning sessions was not associated with a potential lack of effect on retrieval. However, the ACT effect occurred at a higher dose (100 ng/bee) whereas DEL impaired retrieval performance at a lower dose (10 ng/bee). In the same way, when drugs were applied during learning sessions, they strongly impaired retrieval of the PER at 24 h after learning. ACT was the only one able to impair retrieval at a lower dose. Compared to previous studies, the present data suggested that at a lower dose, DEL impaired retrieval when it was applied before the learning session whereas ACT is involved in the retention process when it was administered during learning sessions. These data are consistent with previous studies demonstrating that 12 ng IMI decreased the acquisition process when it was injected 30 min before learning experiment and retention tests 24 h after learning trial [16]. ACT and DEL effects identified in the present study could be due to experimental conditions in which forager bees are collected from the flowers and also to the inter-trial learning delay (2 min, compared to other studies that use 20–30 min). In addition, it is possible that forager bees collected at Orléans (in the present study) are more sensitive than other bees because it was demonstrated that the sublethal effects of a neonicotinoid could be modified by environmental interactions specific to the landscape and time of exposure [19]. Moreover, it is possible to hypothesize that these forager bees collected directly in the flowers are more sensitive to ACT and DEL.

## 5. Conclusions

It seems that compared to control bees, ACT is able to impair retrieval performance 24 h after learning sessions [17]. Thus, the present results are consistent with recent studies demonstrating that neonicotinoids affect honey bee retrieval performance. They confirm the hypothesis that the effect of pesticides on insects is strongly associated with the time of application.
